# A Case of Pharyngeal-Cervical-Brachial Variant of Guillain-Barré Syndrome With Bilateral Glossopharyngeal Paralysis

**DOI:** 10.7759/cureus.14551

**Published:** 2021-04-18

**Authors:** Nancy He, Michael C Kartiko, Mohammad Selim

**Affiliations:** 1 Internal Medicine, Creighton University School of Medicine, Omaha, USA

**Keywords:** bilateral glossopharyngeal paralysis, pharyngeal-cervical-brachial variant, guillain-barre syndrome (gbs)

## Abstract

Guillain-Barré Syndrome (GBS) is a rapidly evolving autoimmune inflammatory disease of the peripheral nerves. It classically presents with progressive symmetrical ascending muscle weakness and hyporeflexia. The pharyngeal-cervical-brachial (PCB) variant is a rare variant of GBS that is characterized by axonal rather than demyelinating neuropathy and presents with rapidly progressive oropharyngeal (facial palsy, dysarthria) and cervicobrachial weakness, associated upper limb weakness, and hyporeflexia. Because it is rare, the PCB variant of GBS is unfamiliar to many neurologists and it is often misdiagnosed as stroke, myasthenia gravis, or botulism. The prevalence of this variant is estimated to be about 3% of all GBS patients. We describe the only known case presentation of the PCB variant of GBS that presents with bilateral glossopharyngeal paralysis. A 39-year-old African American female presented with progressive oropharyngeal and cervicobrachial weakness along with bilateral glossopharyngeal paralysis. The patient was diagnosed based on clinical suspicion, presentation, and serum ganglioside antibodies.

## Introduction

Guillain-Barré Syndrome (GBS) is an autoimmune-mediated peripheral neuropathy characterized by progressive symmetric motor weakness, sensory changes, hyporeflexia, and autonomic symptoms. GBS is most often triggered by an antecedent infection, with the most common pathogen being Campylobacter jejuni though Influenza, Cytomegalovirus, Epstein-Barr virus, and Zika virus have also been implicated. The etiology of GBS is incompletely understood, but the most accepted hypothesis is that molecular mimicry is the immunopathogenic mechanism behind peripheral nerve fiber damage. Diagnosis of GBS is made based on clinical symptoms and confirmed with cerebral spinal fluid (CSF) analysis and nerve conduction studies. CSF testing shows increased protein levels with a normal white blood cell (WBC) count, and nerve conduction studies show conduction slowing or blockage in peripheral nerve fibers. Also, serum anti-ganglioside antibodies are found in some GBS patients. More specifically, patients with pharyngeal-cervical-brachial (PCB) variant carry Immunoglobulin-G (IgG) anti-GT1a antibodies, which often cross-react with GQ1b antibodies.

## Case presentation

A 39-year-old African American female with a past medical history of pseudotumor cerebri, migraine, epilepsy, and hypertension was admitted to the hospital from the ED with dysphagia, slurred speech, tongue numbness, and altered voice. She also endorsed progressive right upper and lower extremity weakness and numbness when compared to the left side. She could walk into the ED but later was unable to stand or move her right leg. The patient exhibited left-sided facial asymmetry with an absence of ptosis, dysarthric speech, and right arm weakness on physical examination. Flexible laryngoscopy showed bilateral soft palate paralysis, absent gag reflex, and poor constrictor function with effort. Other cranial nerves (CNs) were intact. Head CT, computed tomography angiogram (CTA), MRI, lumbar puncture, and electromyography (EMG) were all unremarkable. 

On day 2 of hospitalization, the patient suffered acute hypoxemic respiratory failure due to secretions and inability to protect her airways secondary to pharyngeal paresis. After several attempts, she was successfully intubated. Due to suspicion of a PCB variant of GBS involving pharyngeal and bulbar muscles, serum ganglioside antibodies were sent. The patient began empiric treatment with five sessions of plasmapheresis. Lab results showed high serum GD1a, GD1b, and GQ1b antibodies, IgG-IgM. After three sessions of plasmapheresis, her gag and cough reflexes recovered, and she was successfully extubated. She finished her last two plasmapheresis sessions and was discharged with a referral to neurology for EMG and follow-up on PCB variant of GBS.

## Discussion

GBS is an acute monophasic immune-mediated polyradiculoneuropathy characterized by rapidly ascending weakness, sensory loss, and hyporeflexia [1]. The disease affects all races with a mean age of onset of 40 years and a slight male predominance. The overall incidence was found to be 1.1 to 1.8/100,000. 

The PCB variant of GBS manifests in up to 3% of patients. It is an axonal rather than demyelinating neuropathy [2]. It presents with ptosis, facial, pharyngeal, and neck flexor muscle weakness that spreads to arms with arm areflexia/hyporeflexia [1,2]. It must present in the absence of ataxia, disturbed consciousness, and prominent leg weakness [2]. Sensory symptoms in upper limbs and lower limb weakness may be present but are much less pronounced. The most common initial symptom is arm weakness and the second most common is dysphagia [3]. A total of 81% of patients have a history of an antecedent illness. Approximately half of all GBS patients have CN involvement, with the majority having unilateral or bilateral facial nerve involvement. The next most common involves paralysis of the tongue, lips, mastication muscles, or pharyngeal muscles from lesions involving CNs V, IX, and X [4]. There have been a few reported cases of bilateral CN palsies secondary to GBS. Bilateral facial nerve palsy is a relatively rare GBS presentation but has been observed in some cases [5]. There have also been rare cases of glossopharyngeal nerve palsy secondary to CN ischemia and GBS, especially the PCB variant [6]. However, bilateral involvement of the glossopharyngeal nerve secondary to GBS has never been reported before. Thus far, the only known cases of bilateral glossopharyngeal nerve paralysis have been caused by direct nerve damage as a severe complication of tonsillectomy [4,7]. Bilateral glossopharyngeal paralysis alone is extremely rare and has never been seen before in association with GBS until this current case.

Diagnosis of GBS is made clinically and confirmed with CSF analysis and nerve conduction studies. GBS diagnostic criteria include progressive ascending symmetrical muscle weakness and decreased or absent myotatic reflexes. Once all other causes have been excluded, lumbar puncture should be performed. CSF analysis classically shows albuminocytologic dissociation with elevated protein levels and a normal WBC count [8]. In addition, autoantibodies against specific neuronal gangliosides have been implicated in GBS variants and have been found to be present in at least one-third of patients [8]. In the PCB variant, IgG anti-GT1a and anti-GD1a antibodies have been seen. A clinical study showed that half carried IgG anti-GT1 antibodies, which often cross-reacted with GQ1b, a serological marker of Miller Fisher syndrome [3]. It also revealed positive anti-GQ1b IgG antibodies in 39% of patients. A quarter of patients displayed IgG antibodies against GM1 or GD1a [3]. Due to the rarity of the PCB variant, it is often misdiagnosed as a brainstem stroke. Brain/spinal cord imaging with CT/MRI is not part of the routine diagnostic procedure, but can help exclude other differential diagnoses [9]. 

Plasma exchange and intravenous immunoglobulin (IVIg) are the mainstays of treatment for GBS, with both forms shown to be equally effective [10]. Treatment benefits from IVIg when initiated within two weeks of symptom onset and plasmapheresis within four weeks [10]. Treatment should be considered in patients with rapidly progressive weakness, autonomic dysfunction, bulbar failure, or respiratory insufficiency [10]. Antimicrobial or antiviral therapy should also be considered. However, in most cases, the antecedent infection usually resolves before the onset of motor weakness [10]. Patients with PCB variant are more likely to require intubation due to bulbar involvement and require ongoing assessment of bulbar function and respiratory effort to guide the use of nasogastric feeding and ventilator support [2].

## Conclusions

PCB is a rare variant of GBS that is unfamiliar to many neurologists and often misdiagnosed. We have reported a unique case of a PCB variant of GBS that presented with bilateral glossopharyngeal paralysis. Bilateral glossopharyngeal paralysis is extremely rare and has never been seen before in association with GBS. Early clinical recognition of this presentation in the PCB variant is essential for preventing respiratory-associated morbidity and mortality.
